# SARS-CoV-2 spike protein displays sequence similarities with paramyxovirus surface proteins; a bioinformatics study

**DOI:** 10.1371/journal.pone.0260360

**Published:** 2021-12-02

**Authors:** Ehsan Ahmadi, Mohammad Reza Zabihi, Ramin Hosseinzadeh, Leila Mohamed Khosroshahi, Farshid Noorbakhsh

**Affiliations:** 1 Department of Immunology, School of Medicine, Tehran University of Medical Sciences, Tehran, Iran; 2 Shefa Neuroscience Research Center, Khatam Alanbia Hospital, Tehran, Iran; Waseda University: Waseda Daigaku, JAPAN

## Abstract

Recent emergence of SARS-CoV-2 and associated COVID-19 pandemic have posed a great challenge for the scientific community. In this study, we performed bioinformatic analyses on SARS-CoV-2 protein sequences, trying to unravel potential molecular similarities between this newly emerged pathogen with non-coronavirus ssRNA viruses. Comparing the proteins of SARS-CoV-2 with non-coronavirus positive and negative strand ssRNA viruses revealed multiple sequence similarities between SARS-CoV-2 and non-coronaviruses, including similarities between RNA-dependent RNA-polymerases and helicases (two highly-conserved proteins). We also observed similarities between SARS-CoV-2 surface (i.e. spike) protein with paramyxovirus fusion proteins. This similarity was restricted to a segment of spike protein S2 subunit which is involved in cell fusion. We next analyzed spike proteins from SARS-CoV-2 “variants of concern” (VOCs) and “variants of interests” (VOIs) and found that some of these variants show considerably higher spike-fusion similarity with paramyxoviruses. The ‘spike-fusion’ similarity was also observed for some pathogenic coronaviruses other than SARS-CoV-2. Epitope analysis using experimentally verified data deposited in Immune Epitope Database (IEDB) revealed that several B cell epitopes as well as T cell and MHC binding epitopes map within the spike-fusion similarity region. These data indicate that there might be a degree of convergent evolution between SARS-CoV-2 and paramyxovirus surface proteins which could be of pathogenic and immunological importance.

## Introduction

Current COVID-19 pandemic which is caused by a newly emerged betacoronavirus has led to immense health and socio-economic problems around the world. First human coronaviruses were discovered in 1960s, but human coronavirus infections have likely existed for centuries [1–4]. In the first few decades after their discovery, and when compared with other major viral pathogens like smallpox, polio and influenza, coronaviruses seemed to display a more benign attitude towards their hosts. Nonetheless, emergence of three deadly coronavirus epidemics over the last 20 years has challenged this view and is likely indicative of the dynamic evolution of these pathogens [5, 6]. This necessitates investigations that can provide a better insight regarding coronavirus genes, proteins and biological behavior. One approach towards better understanding of a new pathogen is comparative analysis of its nucleic acids, proteins and molecular structures. The results of these types of analyses can go beyond ordinary phylogenetics and might have applied clinicopathological implications. Indeed, several reports have suggested that immunization against other human pathogens might influence susceptibility to SARS-CoV-2. Viral vaccines including polio and MMR have been suggested as candidates that might give rise to this potential ‘cross-protective’ phenomenon, raising the possibility for the existence of similar molecular structures [7–10].

SARS-CoV-2 nucleic acid and protein sequences did become available shortly after the start of COVID-19 pandemic and biological aspects of these genes/proteins including their interactions with immune system have been under intense investigation. In this study, we performed a comprehensive bioinformatic analysis on SARS-CoV-2 protein sequences to determine whether there might be any biologically interesting similarities between SARS-CoV-2 proteins with non-coronavirus ssRNA viruses. We performed protein similarity searches for individual SARS-CoV-2 proteins against available protein sequences from different ssRNA virus families. This revealed some interesting similarities between SARS-CoV-2 spike protein and paramyxovirus surface proteins as well as SARS-CoV-2 RdRp (RNA-dependent RNA polymerase) and helicase proteins with Togaviruses and Caliciviruses. Considering the importance of spike proteins in viral infectivity and immune response, we then performed more analyses on other coronavirus spike proteins as well as analyses to determine potential immunological significance of these similarities.

## Methods

### Extracting SARS-CoV-2 and other coronavirus protein sequences

SARS-CoV-2 Reference Protein sequences were obtained from NCBI Protein database (https://www.ncbi.nlm.nih.gov/protein). Redundant sequences as well as polyprotein sequences (i.e. ORF1ab and ORF1a) were removed. RefSeq accession numbers and their official gene names are shown in S1 Table in S1 File. Sequences for the surface proteins of various human coronaviruses were also obtained from NCBI Protein database (S2 Table in S1 File). Surface protein sequence from SARS-CoV-2 ‘variants of concern’ (VOC) and ‘variants of interest’ (VOI) were obtained from ViralZone (https://viralzone.expasy.org).

### Protein sequence similarity searches

Protein sequence similarity searches were performed for all SARS-CoV-2 proteins against positive and negative strand ssRNA virus families using Delta-BLAST algorithm. All Delta-BLASTs were followed by three iterations of PSI-BLAST. To detect and report the strongest similarities, best E value-alignment score pairs were extracted for each query. Likewise, protein similarity searches were performed for spike proteins of other human coronaviruses against ssRNA virus families. Results were shown as negative log10 of E values and alignment scores. All of Delta-BLAST parameters are shown in S3 Table in S1 File.

Virus-Host database (https://www.genome.jp/virushostdb/) was used to extract the list of viruses which are pathogenic for humans (i.e. pathogenic Togaviridae, Caliciviridae and Paramyxoviridae).

### Determining SARS-CoV-2 spike protein epitopes and their distribution

Peptide sequences from SARS-CoV-2 spike protein which could act as B cell or T cell epitopes or MHC binders were determined using immune epitope database (IEDB). Epitope search was limited to experimentally verified epitopes. Linear epitopes were considered that had exact matches with substrings of SARS-CoV-2 spike protein sequence. Pairwise BLASTP search was used to find the distribution of these epitopes on spike protein.

### Phylogenetic analysis

Phylogenetic analysis was performed using RNA-dependent RNA polymerase (RdRP) protein sequences from different ssRNA viruses. RdRP sequences from representative positive strand and negative strand ssRNA viruses were obtained from NCBI Protein Database. Multiple sequence alignment (MSA) and phylogenetic analysis were performed using MEGA X and PhyML [11]. MUSCLE algorithm was used to perform MSA between sequences. Maximum likelihood (ML) model selection was performed using “Smart Model Selection in PhyML” (SMS) [12]. Phylogenetic tree construction was performed in PhyML and MEGAX. Generated trees were tested by 100X bootstrapping.

## Results

### Comparing SARS-CoV-2 protein sequences with other ssRNA viruses

**Fig 1:**
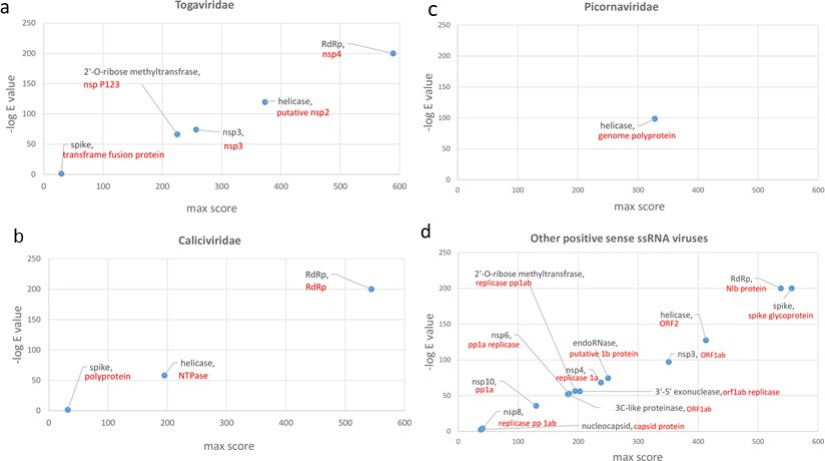
Sequence similarity between SARS-CoV-2 proteins and positive sense ssRNA virus families. Results of sequence similarity searches for 25 SARS-CoV-2 RefSeq proteins against positive strand ssRNA viruses are shown as scatter plots. X axis in each graph shows the maximum score obtained for each protein query. Y axis in each graph shows the highest negative log E-value obtained for each protein query. Black labels show the name of SARS-CoV-2 protein. Red labels show the name of the protein from the relevant ssRNA virus family. The name of virus species with sequence similarity is included in S4 Table in S1 File. Abbreviations: NSP, non-structural protein; RdRp, RNA-dependent RNA polymerase.

SARS-CoV-2 is a member of betacoronaviruses, which belong to Nidovirales order of positive sense ssRNA viruses [13]. To investigate potential molecular similarities between this virus and other ssRNA viruses, we first compared all of SARS-CoV-2 proteins (25 RefSeq proteins, S1 Table in S1 File) with protein sequences from various positive strand ssRNA virus families. To have a more clear view, we first analyzed virus families which include major human pathogens, i.e. Picornaviridae, Flaviviridae, Togaviridae and Caliciviridae. As described in the Methods section, Delta-BLAST followed by thee PSI-BLAST iterations were used to perform the comparisons and best E value/alignment scores were determined for each SARS-CoV-2 protein. As shown in Fig 1, similarity searches showed significant similarities (E values below 1E-200) between SARS-CoV-2 RdRp with Togaviridae and Caliciviridae RdRp proteins (Fig 1A and 1B and S4 Table in S1 File). Less significant similarities were observed between SARS-CoV-2 helicase (E value around 1E-120) with Togaviridae, Caliciviridae and Picornaviridae helicases (Fig 1A–1C and S4 Table in S1 File). No significant similarities could be detected with members of Flaviviridae. We also performed similarity searches for other positive sense ssRNA virus families, which showed the similar findings for RdRp as well as helicase (Fig 1D and S4 Table in S1 File). These findings were expectable considering the highly conserved nature of these enzymes. Of note, SARS-CoV2 surface protein showed a high degree of alignment with spike proteins from “unclassified nidovirales”, which is likely a reflection of phylogenetic relationship (Fig 1D).

**Fig 2:**
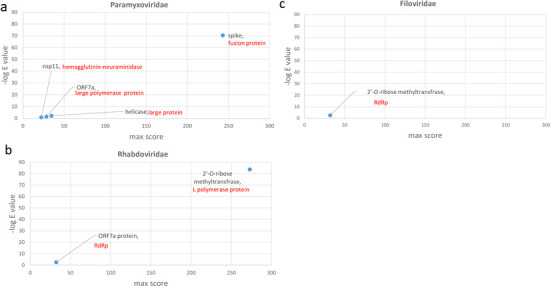
Sequence similarity between SARS-CoV-2 proteins and negative sense ssRNA virus families. Results of similarity searches for 25 SARS-CoV-2 RefSeq proteins against negative strand ssRNA viruses are shown as scatter plots. X axis in each graph shows the maximum score obtained for each protein query. Y axis in each graph shows the highest negative log E-value obtained for each protein query. Black labels show the name of SARS-CoV-2 protein. Red labels show the name of the protein from the relevant ssRNA virus family. The name of virus species with sequence similarity is included in S5 Table in S1 File.

We next compared SARS-CoV-2 proteins with negative strand ssRNA viruses, with Paramyxoviridae, Orthomyxoviridae, Rhabdoviridae and Filoviridae, analyzed individually. Similarity searches against Paramyxoviridae showed similarities between SARS-CoV-2 spike protein and fusion proteins of various paramyxoviruses (E value around 4E-70) (Fig 2A and S5 Table in S1 File). Sequence comparisons with for Rhabdoviridae showed similarities between SARS-CoV-2 2’-O-ribose methyltransferase with ‘L polymerase protein’ in Rhabdoviruses (Fig 2B and S5 Table in S1 File). Comparison with Filoviridae showed very limited similarities (Fig 2C); no significant similarities were observed for Orthomyxoviridae or any other negative sense ssRNA families.

**Fig 3:**
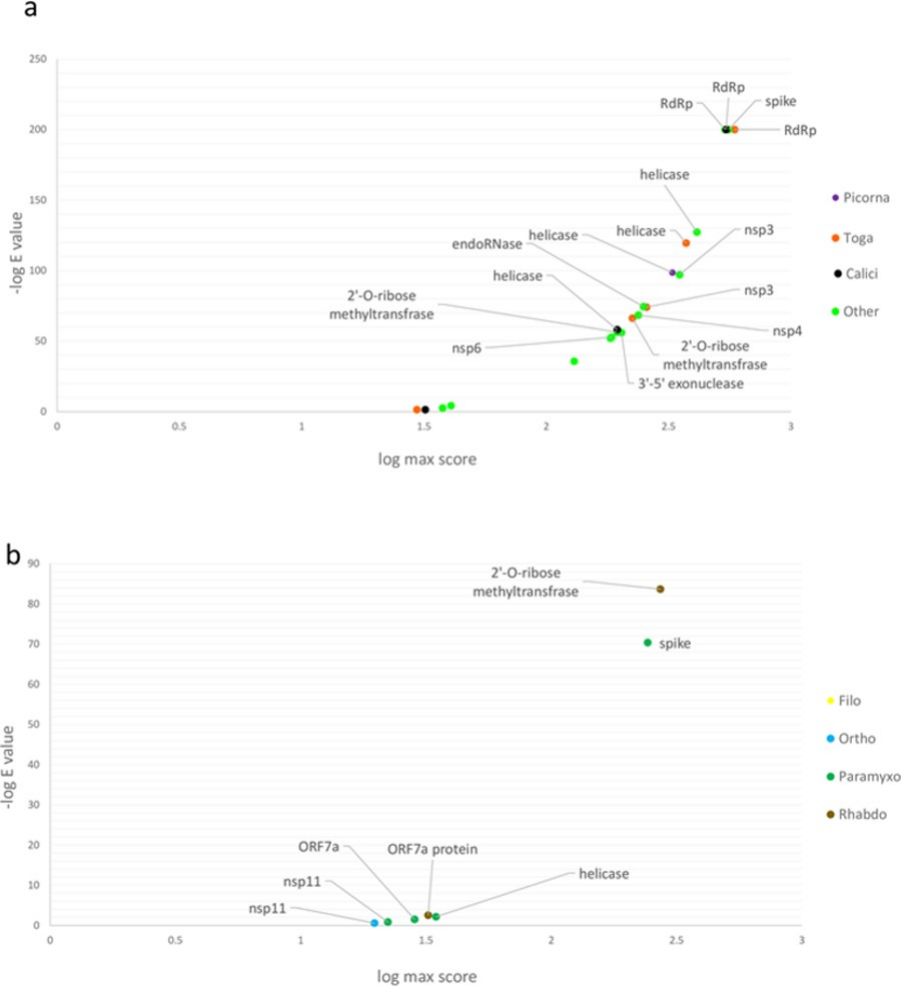
Overlay plots for SARS-CoV2’s most significant protein similarities with non-coronavirus ssRNA viruses. Score-negative log E value for various SARS-CoV-2 proteins against positive sense ssRNA virus families (a) and negative sense ssRNA virus families (b). For each protein query, only the highest score-negative log E value pair is shown.

Results of all similarity searches for positive and negative sense ssRNA viruses are illustrated in overlay plots (Fig 3A and 3B). Further comparisons between SARS-CoV-2 RdRp and pathogenic human Togaviridae and Caliciviridae reveled the highest degrees of similarity with Sindbis virus and noroviruses, respectively (S1 and S2 Figs in S1 File). Nonetheless, considering the importance of viral surface proteins in receptor binding and cell entry, we decided to focus and further explore the sequence similarity between SARS-CoV-2 surface proteins with paramyxovirus proteins.

**Fig 4:**
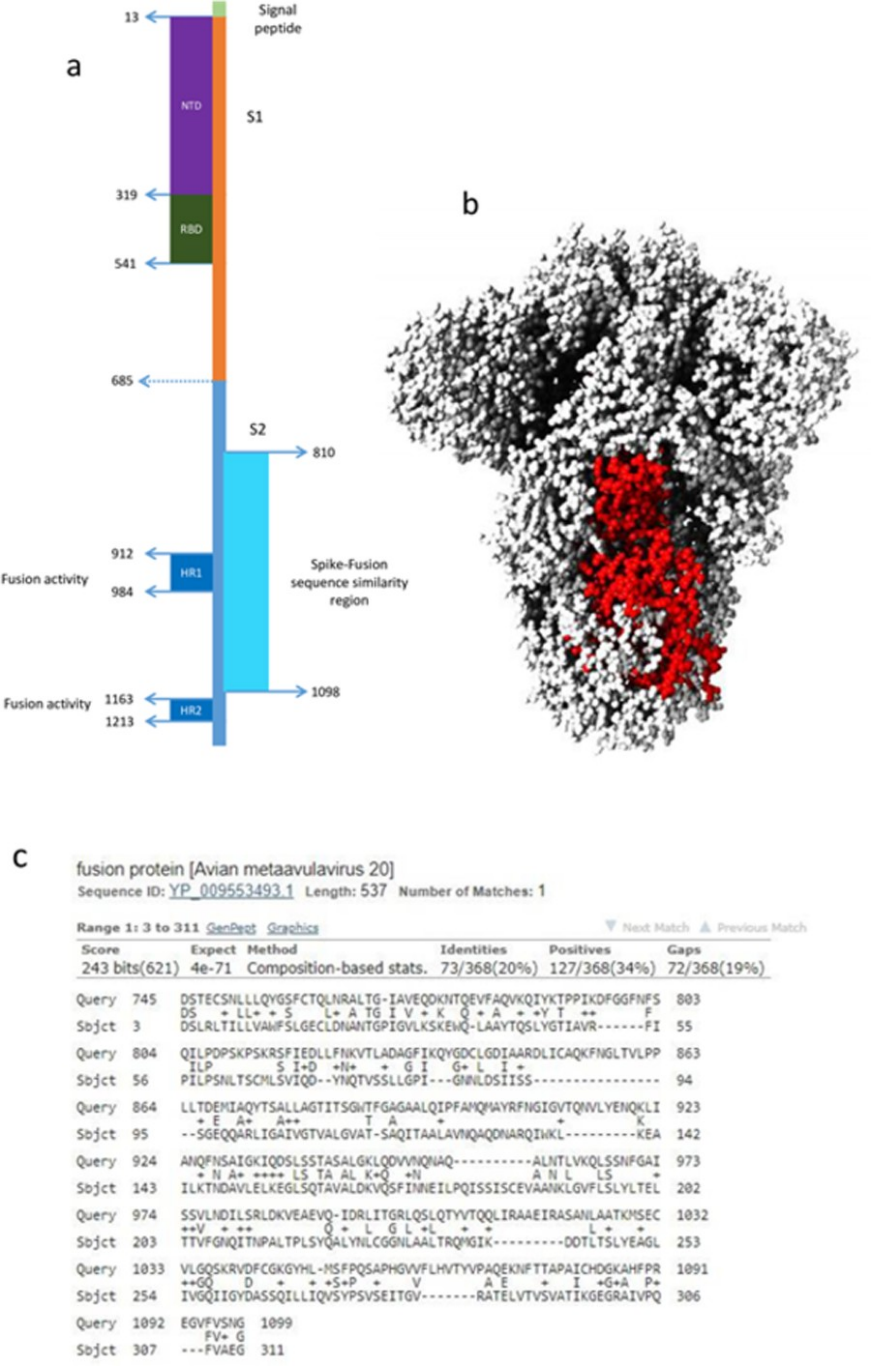
Spike-Fusion protein similarity region on spike protein. SARS-CoV-2 spike schematic model with paramyxovirus sequence similarity region shown with pale blue bar (residues 810 to 1098) (a). Corresponding area is shown in red on SARS-CoV-2 spike 3D structure (b). Delta-BLAST result for SARS-CoV-2 spike protein versus Avian metaavulavirus 20 fusion protein, which showed the highest score-negative E value in Delta-BLAST searches (c).

**Fig 5:**
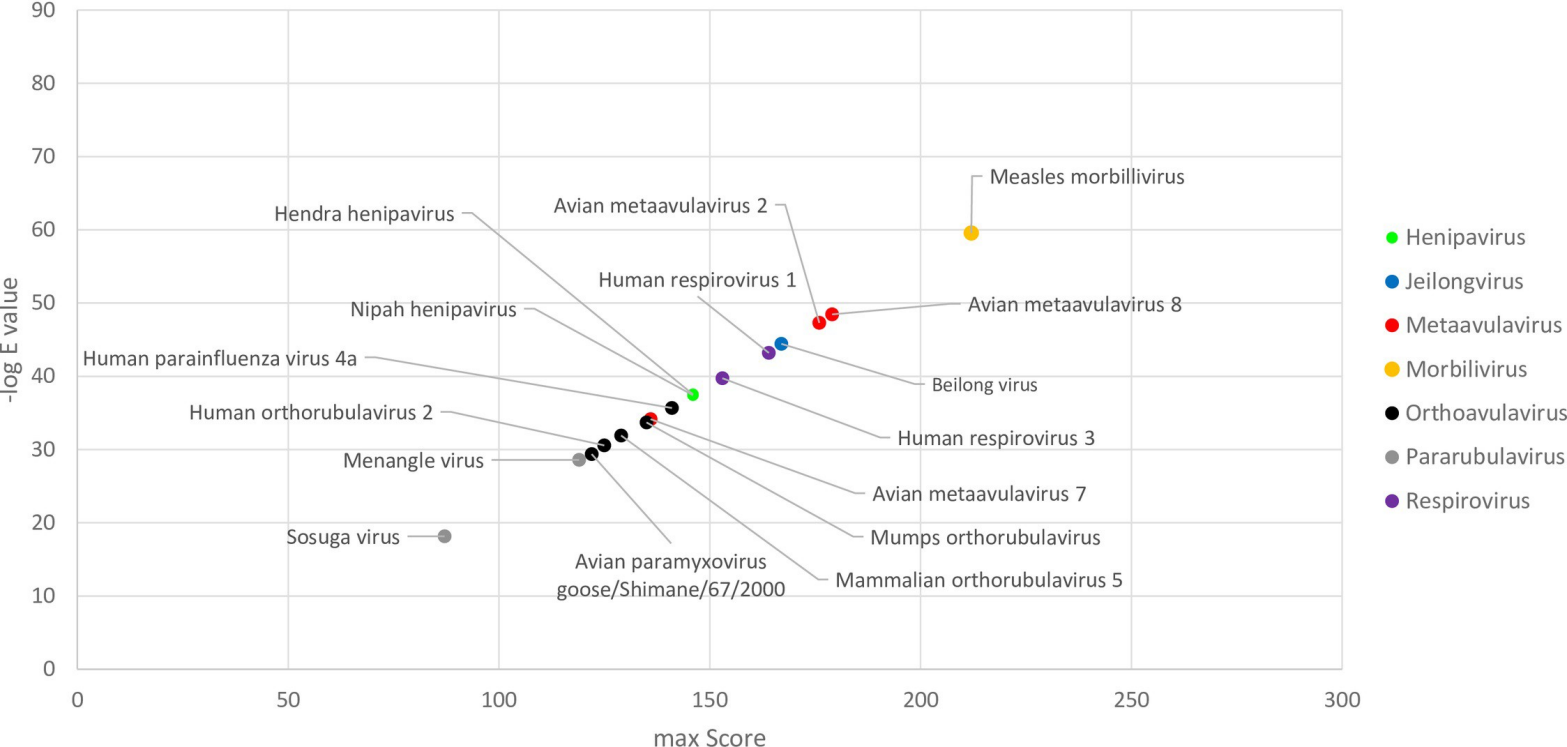
SARS-CoV-2 spike protein similarity with pathogenic human paramyxoviruses. The result of similarity searches between SARS-CoV-2 spike protein versus different human paramyxoviruses. The X axis shows maximum score for each protein query and the Y axis shows negative log E value for each protein query. Dot colors and legend show different genera of paramyxoviruses.

The region of SARS-CoV-2 spike protein which shows similarity with paramyxovirus fusion proteins in displayed in Fig 4. This is a segment of SARS-CoV-2 spike protein S2 subunit which is involved in cell fusion [14]. Based on their structural features, viral fusion proteins are categorized into four classes; Type I to IV [15, 16]. Coronavirus and paramyxovirus fusion proteins belong to Class I; these are trimeric proteins which contain both a receptor-binding domain and a fusion domain [15, 16]. It is conceivable that type I fusion proteins demonstrate a degree of sequence similarity; however, SARS-CoV-2 spike did not show any sequence similarity with fusion proteins from Orthomyxoviridae or Filoviridae which also have Type I fusion proteins. To gain a better insight into the observed SARS-Cov-2-paramyxovirus protein similarity (hereafter called ‘spike-fusion’ similarity), we obtained a list of pathogenic human paramyxoviruses from Virus-Host database (genome.jp/virushostdb/) and extracted their alignment score- E values from Delta-BLAST search. As shown in Fig 5, measles morbillivirus followed by metaavulaviruses and respiviruses revealed highest degrees of similarity.

### Different SARS-CoV-2 and coronavirus spike proteins show different degrees of similarity with paramyxoviruses

**Fig 6:**
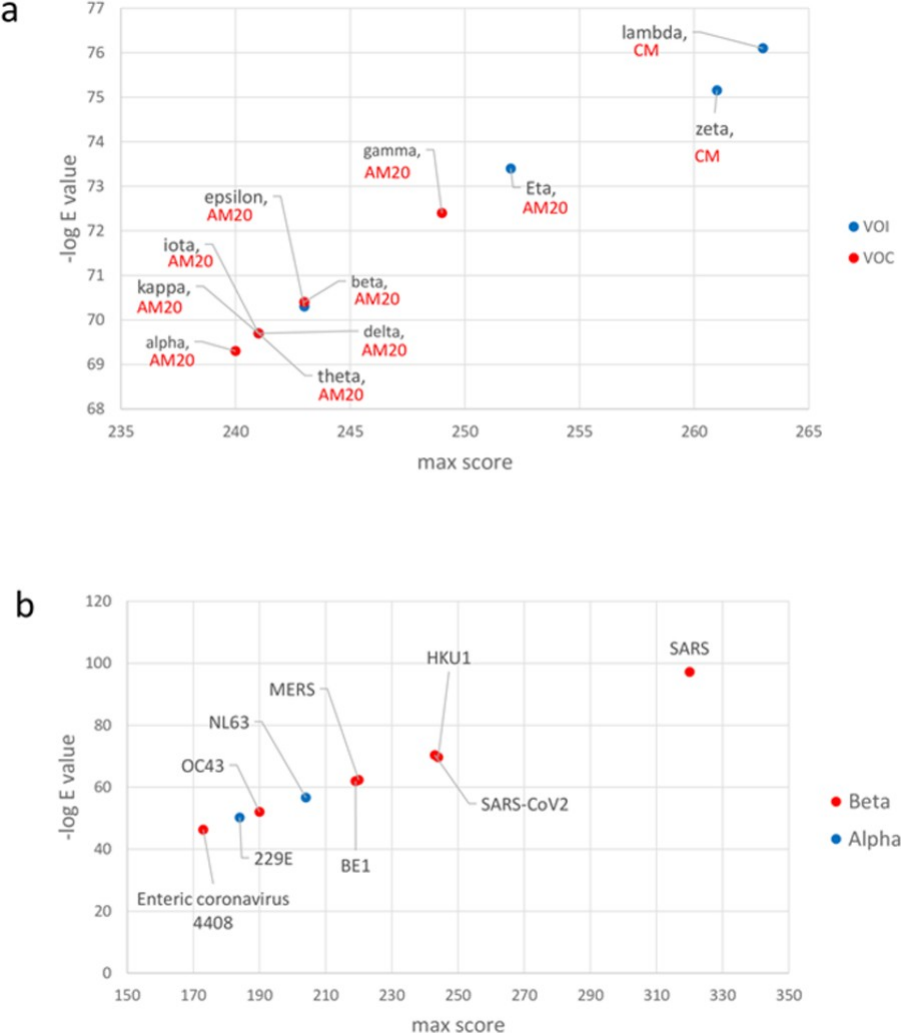
Spike similarity between different variants of SARS-CoV-2 and different subgroups of coronaviruses versus fusion protein of paramyxoviruses. Sequence similarity between VOI and VOC variants of SARS-CoV-2 with fusion proteins of Paramyxoviruses (a). AM20 is avian metaavulavirus 20. CM is Canine morbillivirus. Spike similarity of alpha (blue dots) and beta (red dots) coronaviruses with fusion proteins of Paramyxoviruses (b). The X axis shows maximum score and the Y axis shows negative log of E value in each graph.

Since its discovery in late 2019, SARS-CoV-2 has been undergoing genetic changes that have influenced its transmissibility and pathogenesis. Based on criteria including their virulence, transmissibility and susceptibility to antiviral drugs, SARS-CoV-2 variants have been broadly categorized to ‘variants of concern’ (VOC) and ‘variants of interest’ (VOI) [17]. Various mutations have been reported in the spike protein in SARS-COV2 variants, and while the majority of these mutations are located in the receptor binding domain (RBD) or the N-terminal domain (NTD) of spike protein, some amino acid changes have also been detected in the S2 region, where the observed sequence similarities with paramyxoviruses exists. We compared the spike sequences from several SARS-CoV-2 variants with paramyxovirus proteins. As shown in Fig 6A, it seems that variants lambda and zeta show slightly higher degrees of similarities with paramyxovirus fusion proteins. This is followed by alpha to delta variants. Highest similarity Score-E values were seen for canine morbillivirus and avian metaavulavirus fusion proteins.

We next asked whether the observed similarity between spike protein and paramyxovirus fusion proteins is limited to SARS-CoV-2 or that it is a general phenomenon among coronaviruses. To answer this question, we compiled a list of human coronaviruses using Virus-Host database (https://www.genome.jp/virushostdb) and obtained their spike protein sequences from NCBI protein database (S2 Table in S1 File). We next performed Delta-BLAST searches for each of these spike proteins against Paramyxoviridae protein sequences. Our analyses showed that Spike-Fusion similarities do exist for some other human coronaviruses (Fig 6B). Interestingly, two highly pathogenic coronaviruses (i.e. SARS-CoV and SARS-CoV2) showed a much higher degree of similarity with paramyxovirus surface proteins, compared with similarities observed for other betacoronaviruses. HKU1 human coronavirus had scores-log E values similar with SARS-CoV-2. Less pathogenic coronaviruses including OC43 betacoronavirus and NL63 and 229E alphacoronaviruses displayed spike sequence similarities with paramyxoviridae, albeit with much lower score-log E values (Fig 6B). Coronaviruses express three proteins at their surface; S (spike), E (envelope) and M (membrane). To determine whether similarities might also exist for the latter two proteins, we performed similar analyses for all coronavirus E and M proteins against paramyxoviruses. No significant similarities with paramyxoviruses were observed for either E or M proteins.

### Spike-paramyxovirus similarity regions might bear immunological significance

**Fig 7:**
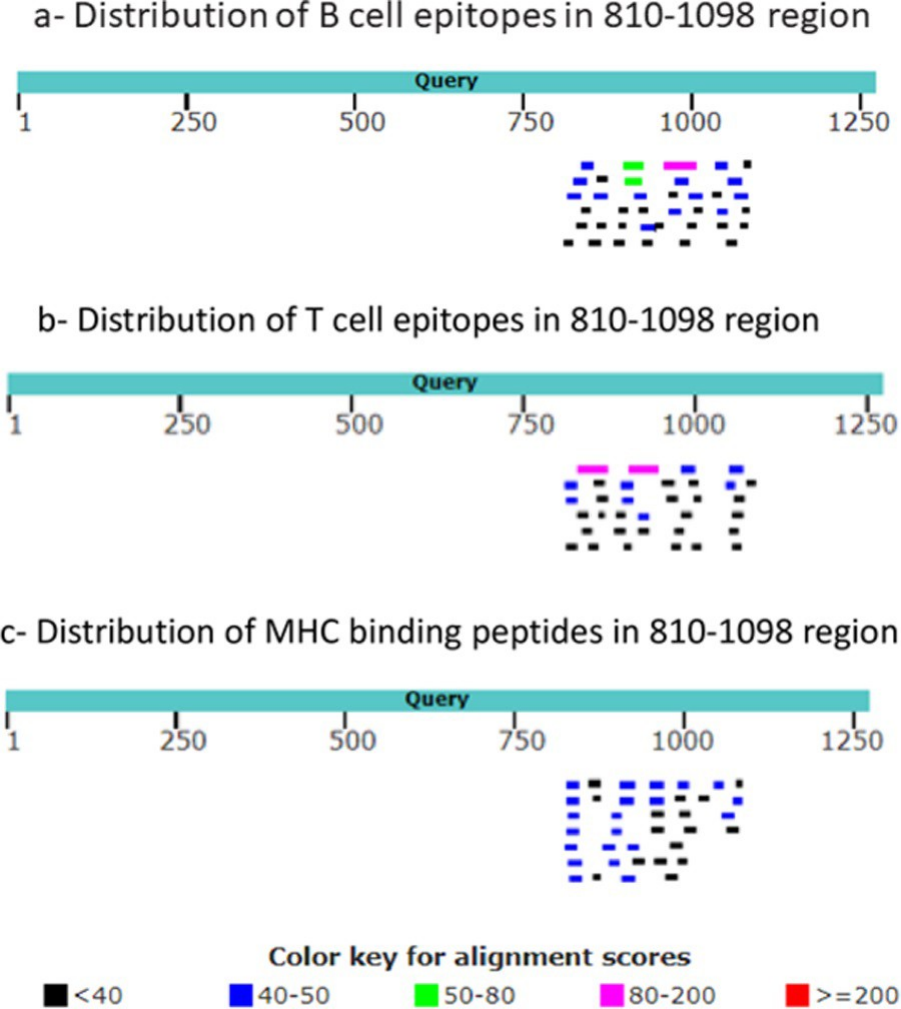
Distribution of B cell and T cell epitopes as well MHC binding peptides inside spike-fusion similarity region. Experimentally verified B cell epitopes (a), T cell epitopes (B) and MHC binding peptides (c) which matched substrings of SARS-CoV2 spike protein were obtained from IEDB and aligned with SARS-CoV2 spike protein to determine their distribution across the area.

Several reports have raised the possibility that immune response generated by MMR vaccine might have a protective effect against COVID-19 [18–23]. A study by Gold et al has shown a negative inverse correlation between mumps antibody titers with COVID-19 severity [18]. Another study has reported that heterologous T cell immunity might be involved in MMR-COVID-19 cross-protective effects [24]. A homology modeling by Marakasova et al has revealed a region of similarity between SARS-CoV-2 RBD with measles fusion protein [25]. Moreover, an *in silico* analysis to find similar segments between antigenic proteins of various vaccines with SARS-CoV-2 proteins has detected two short stretches of amino acids which are common between measles fusion proteins and SARS-CoV2 ORF6 and ORF8, and between mumps fusion protein and SARS-CoV-2 spike amino acids 467 to 473 [26]. Considering these reports, we asked whether sequence similarity that we found between SARS-COV-2 spike and paramyxovirus fusion proteins might have any immunological importance. We searched Immune Epitope Database (IEDB) for experimentally verified B cell and T cell epitopes which exist in SARS-CoV-2 spike protein between amino acids 810 to 1098. As shown in Fig 7, several verified B cell and T cell epitopes as well MHC binding peptides could be detected in this region. Of interest, three of the B cell epitopes were associated with neutralizing antibodies (S6 Table in S1 File). Likewise, several of the T cell epitopes were linked with cytokine production (S7 Table in S1 File). These data imply that spike-fusion region of similarity might have some immunological importance. Nonetheless, whether the presence of these epitopes in spike-fusion similarity region does indeed lead to any cross-reactivity/cross-protection between antibodies raised by MMR vaccines and SARS-CoV-2 would require experimental studies.

### Observed SARS-CoV-2 spike-fusion similarities do not follow the general pattern of ssRNA virus phylogenetic relations

**Fig 8:**
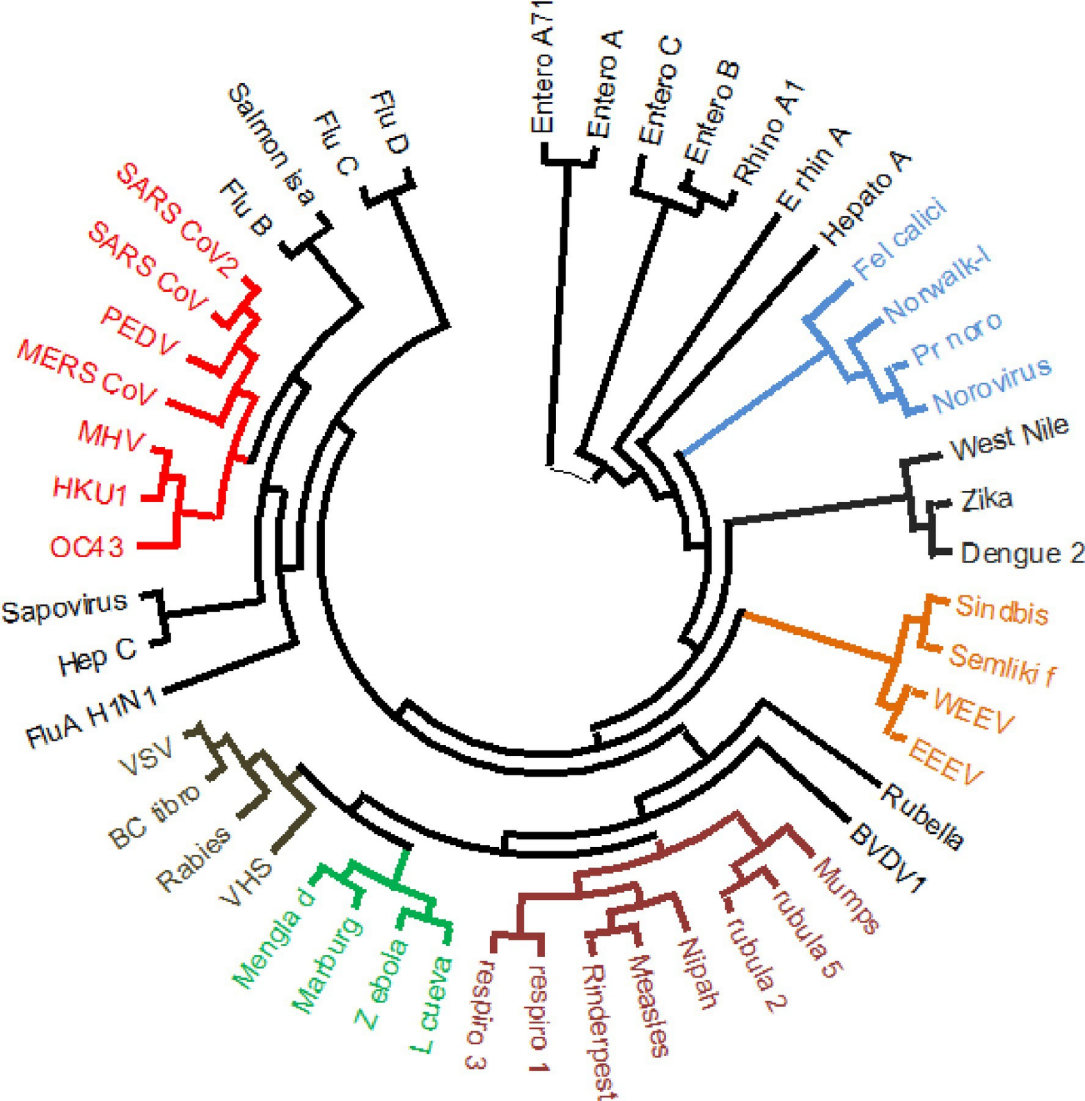
Phylogenetic tree of ssRNA viruses. Phylogenetic tree of representative members of positive sense strand ssRNA viruses (i.e. Picornaviridae, Caliciviridae, Coronaviridae, Flaviviridae and Togaviridae) and negative sense strand ssRNA viruses (i.e. Orthomyxoviridae, Paramyxoviridae, Filoviridae, Rhabdoviridae) was drawn based on viral RdRP protein sequences. Virus name abbreviations: BC tibro, Bas-Congo tibrovirus; BVDV1, Bovine viral diarrhea virus 1; EEEV, Eastern equine encephalitis virus; L Cueva, Lloviu cuevavirus; PEDV, Porcine epidemic diarrhea virus; Pr-nor, Primate norovirus; Semliki F, Semliki Forest virus; VHS, Viral hemorrhagic septicemia virus; VSV, Vesicular stomatitis Indiana virus; WEEV, Western equine encephalitis virus.

The most likely explanation for similarities between nucleic acid or protein sequences from various microorganisms is ‘shared ancestry’. Indeed, the expression ‘sequence homology’ is refereed to those sequence similarities which are a consequence of ‘shared ancestry’. To examine whether observed similarities between SARS-CoV-2 spike and paramyxovirus fusion proteins are a consequence of shared ancestry, we performed phylogenetic analyses on various members of ssRNA viruses. The protein sequence of RNA-dependent RNA polymerase (RdRP) is widely used for performing phylogenetic studies on RNA viruses. Representative pathogenic viruses were selected from each group (shown in S8 Table in S1 File) and relevant RdRP sequences were obtained from NCBI Protein Database. Phylogenetic analysis using Maximum Likelihood method showed separate clusters for the majority of positive strand ssRNA versus negative strand ssRNA viruses (Fig 8). Of note, members of Coronaviridae were clustered separately from Paramyxoviridae based on RdRP protein sequences. This indicated that observed similarity between spike-surface proteins was not necessarily a reflection of general phylogenetic relations, at least when these relations are inferred from RdRp-derived phylogenetic trees.

## Discussion

Microbial pathogens display different degrees of likeness at the level of their macromolecules, as well their biological behavior. This is generally a consequence of shared ancestry, horizontal gene transfers or convergent evolution. When it comes to infectious diseases, these similarities might bear epidemiological or clinical importance. This is especially so when a new pathogen appears, whose evolution, pathogenesis and immunology is largely unknown. In this bioinformatic study, we compared all of SARS-CoV2 proteins with non-coronavirus ssRNA viruses. In addition to similarities between highly conserved proteins like RdRp and helicase, we found a potentially important similarity between SARS-CoV-2 spike protein and fusion proteins of paramyxoviridae. Although this similarity also existed for some other pathogenic betacoronaviruses, similarity observed for SARS-CoV-2 (as well as SARS-CoV) demonstrated a higher score-log E value compared to other human coronaviruses. The degree of similarity differed between SARS-CoV-2 genetic variants and included several B cell, T cell and MHC binding epitopes.

Paramyxoviruses include several major human and animal pathogens, with well-recognized pathophysiology and immune behavior [27–30]. Henipaviruses, morbilliviruses, respiroviruses, and Orthorubulaviruses are known human paramyxoviruses. In our analyses, avian metaavulavirus 20 revealed the highest degree of similarity with SARS-CoV-2 spike protein. Among human pathogens, highest similarities were seen with fusion proteins from measles virus followed by metaavulaviruses and respiroviruses. The observed spike-fusion protein similarity was not limited to SARS-CoV-2. Spike proteins from other betacoronaviruses as well as some alphacoronaviruses also showed some levels of similarity. However, when compared in terms of expect value and alignment score, SARS-CoV and SARS-CoV-2 spike-fusion similarity were much stronger than other pathogenic human betacoronaviruses, i.e. MERS-CoV, or the benign beta or alphacoronaviruses, i.e. OC43, NL63 and 229E.

Evolution from a common ancestor is usually the underlying reason for the existence of similarities between nucleic acid and/or protein sequences. Indeed, the concept of “sequence homology” entails “shared ancestry” and sequence similarities between taxa which do not share a recent common ancestor are referred to as ‘homoplasy’ [31, 32]. Phylogenetic relations of RNA viruses are usually inferred based on analyses of well conserved proteins including RNA polymerases. RdRP-based phylogenetic analysis of representative members of ssRNA viruses showed that the observed Spike-Fusion protein similarity did not seem to follow the branching pattern of inferred phylogenetic tree. While various RNA viruses ought to share a common ancestor at some level of evolution, our current analysis suggests that factors other than shared ancestry might be involved in the observed sequence similarities. SARS-CoV-2 spike and paramyxovirus fusion proteins both belong to type I viral fusion proteins; nonetheless, type I fusion protein also include orthomyxovirus and filovirus fusion proteins, with which SARS-CoV-2 spike did not show any similarities. Considering these matters, we believe that there might be a degree of convergent evolution between SARS-CoV-2 spike and paramyxovirus fusion proteins, which might be driven by selection pressure from host-pathogen interactions. When analyzed for the presence of experimentally verified B cell and/or cell epitopes Spike-Fusion similarity region showed several of these epitopes, compared with the rest the protein sequence. As alluded to before, some recent reports have suggested that vaccination using live MMR vaccine might offer a degree of protection against COVID-19 infection [18–24]. It should be noted that, while antigenic similarities raise the possibility of cross-protective adaptive immune responses, this does not exclude the possibility of involvement of innate immune mechanisms, as shared pathogenic molecular structures might also lead to similar patterns of innate immune activation.

Unraveling different aspects of the evolution of microbial pathogens is of paramount importance in predicting, understanding and controlling infectious disease epidemics. Ordinary bioinformatic analyses on nucleic acid and/or proteins might give us some hints about the relatedness or dissimilarities between pathogenic microorganisms, but grasping the complexity of this dynamism would likely require integrated analyses of host(s)-pathogen(s) co-evolution.

## Supporting information

S1 File (DOCX)
