# Supplementary material for: SARS-CoV-2 spike protein displays sequence similarities with paramyxovirus surface proteins; a bioinformatics study
Source: PLoS One. 2021 Dec 2;16(12):e0260360. doi: 10.1371/journal.pone.0260360 (PMC8639087; doi:10.1371/journal.pone.0260360)
Supplement: S1 File — (DOCX) [file pone.0260360.s001.docx]

**SARS-CoV-2 spike protein displays sequence similarities with paramyxovirus surface proteins; a bioinformatics study**

**--------------------------------------------------------------------------------------------------**

**SARS-CoV2 RdRp protein sequence similarity with pathogenic human Togaviruses**

S1 Fig

S2 Fig

**SARS-CoV2 RdRp protein sequence similarity with pathogenic human Caliciviruses**

**S1 Table- SARS-CoV2 protein names and RefSeq accession numbers**

| No | Name | Accession Numbers | No | Name | Accession Numbers |
| --- | --- | --- | --- | --- | --- |
| 1 | nsp2 | YP_009725298.1, YP_009742609.1 | 14 | endoRnase | YP_009725310.1 |
| 2 | nsp3 | YP_009725299.1, YP_009742610.1 | 15 | 2’-O-ribose methyltransfrase | YP_009725311.1 |
| 3 | nsp4 | YP_009725300.1, YP_009742611.1 | 16 | spike | YP_009724390.1 |
| 4 | 3C-like proteinase | YP_009725301.1, YP_009742612.1 | 17 | ORF3a protein | YP_009724391.1 |
| 5 | nsp6 | YP_009725302.1, YP_009742613.1 | 18 | envelope | YP_009724392.1 |
| 6 | nsp7 | YP_009725303.1, YP_009742614.1 | 19 | membrane | YP_009724393.1 |
| 7 | nsp8 | YP_009725304.1, YP_009742615.1 | 20 | ORF6 | YP_009724394.1 |
| 8 | nsp9 | YP_009725305.1, YP_009742616.1 | 21 | ORF7a | YP_009724395.1 |
| 9 | nsp10 | YP_009725306.1, YP_009742617.1 | 22 | ORF7b | YP_009725318.1 |
| 10 | nsp11 | YP_009725312.1 | 23 | ORF8 | YP_009724396.1 |
| 11 | RdRp | YP_009725307.1 | 24 | nucleocapsid | YP_009724397.2 |
| 12 | helicase | YP_009725308.1 | 25 | ORF10 | YP_009725255.1 |
| 13 | 3’-5’ exonuclease | YP_009725309.1 |  |  |  |

**S2 Table- Spike protein accession numbers for various human Coronaviruses**

| Virus name | Genus | Spike accession number |
| --- | --- | --- |
| SARS | Betacoronavirus | NP_828851.1 |
| SARS-CoV2 | Betacoronavirus | YP_009724390.1 |
| HKU1 | Betacoronavirus | YP_173238.1 |
| MERS | Betacoronavirus | YP_009047204.1 |
| BE1 | Betacoronavirus | YP_007188579.1 |
| OC43 | Betacoronavirus | YP_009555241.1 |
| Enteric coronavirus 4408 | Betacoronavirus | ACT11030.1 |
| NL63 | Alphacoronavirus | YP_003767.1 |
| 229E | Alphacoronavirus | NP_073551.1 |

**S3 Table- Delta-BLAST parameters – Continued on the next page**

| Parameter | Fig1a | Fig1b | Fig1c | Fig1d |
| --- | --- | --- | --- | --- |
| **Database** | RefSeq Proteins | RefSeq Proteins | RefSeq Proteins | RefSeq Proteins |
| **Organism** | Togaviridae | Caliciviridae | Picornaviridae | Pisuviricota Excluding Toagviriade,  Caliciviridae, Picronaviridae,  Durnavirales, Coronaviridae |
| **Program** | Delta-BLAST | Delta-BLAST | Delta-BLAST | Delta-BLAST |
| **Word size** | 3 | 3 | 3 | 3 |
| **Hitlist size** | 500 | 500 | 500 | 500 |
| **Gap Costs** | Existence:11  Extension:1 | Existence:11  Extension:1 | Existence:11  Extension:1 | Existence:11  Extension:1 |
| **Matrix** | BLOSUM62 | BLOSUM62 | BLOSUM62 | BLOSUM62 |
| **PSI-BLAST iterations** | 3 | 3 | 3 | 3 |
| **PSI-BLAST Threshold** | 0.005 | 0.005 | 0.005 | 0.005 |
| **Delta-BLAST Threshold** | 0.05 | 0.05 | 0.05 | 0.05 |

**S3 Table– Continued**

| Parameter | Fig2a | Fig2b | Fig2c | Other negative sense ssRNA |
| --- | --- | --- | --- | --- |
| **Database** | RefSeq Proteins | RefSeq Proteins | RefSeq Proteins | RefSeq Proteins |
| **Organism** | Paramyxoviridae | Rhabdoviridae | Filoviridae | Negarnaviricota Excluding Paramyxoviridae, Rhaboviridae, Filoviridae, Orthomyxoviridae |
| **Program** | Delta-BLAST | Delta-BLAST | Delta-BLAST | Delta-BLAST |
| **Word size** | 3 | 3 | 3 | 3 |
| **Hitlist size** | 500 | 500 | 500 | 500 |
| **Gap Costs** | Existence:11  Extension:1 | Existence:11  Extension:1 | Existence:11  Extension:1 | Existence:11  Extension:1 |
| **Matrix** | BLOSUM62 | BLOSUM62 | BLOSUM62 | BLOSUM62 |
| **PSI-BLAST iterations** | 3 | 3 | 3 | 3 |
| **PSI-BLAST Threshold** | 0.005 | 0.005 | 0.005 | 0.005 |
| **Delta-BLAST Threshold** | 0.05 | 0.05 | 0.05 | 0.05 |

**S4 Table - Delta-BLAST results for positive-sense ssRNA viruses**

| SARS-CoV-2 | Togaviridae | Virus name |
| --- | --- | --- |
| RdRp | nsp4 | Aura virus |
| helicase | putative nsp2 | Venezuelan equine encephalitis virus |
| nsp3 | nsp3 | Cabassou virus |
| 2’-O-ribose methyltransfrase | non structural protein P123 | Semliki forest virus |
| Spike | Transframe fusion protein | Southern elephant seal virus |

| SARS-CoV-2 | Caliciviridae | Virus name |
| --- | --- | --- |
| RdRp | RdRp | Norovirus GII |
| helicase | NTPase | Norovirus GII |
| Spike | polyprotein | Rabbit hemorrhagic disease virus |

| SARS-CoV-2 | Picornaviridae | Virus name |
| --- | --- | --- |
| helicase | genome polyprotein | Enterovirus E |

**S4 Table –Continued**

| SARS-CoV-2 | Other positive sense ssRNA viruses | Virus name |
| --- | --- | --- |
| RdRp | Nlb protein | Lettuce mosaic virus |
| Spike | Spike glycoprotein | Ball python nidovirus 1 |
| helicase | ORF2 | Dianke virus |
| nsp3 | 1ab | Xinzhou toro-like virus |
| endoRNase | putative 1b protein | Mikumi yellow baboon virus 1 |
| nsp4 | replicase 1a | Shingleback nidovirus 1 |
| 2'-O-ribose methyltransfrase | replicase 1b | Shingleback nidovirus 1 |
| 3'-5' exonuclease | ORF1ab replicase polyprotein pp1ab | Gill-associated virus |
| nsp6 | Pp1a replicase polyprotein | Ball python nidovirus 1 |
| 3C-like proteinase | orf1a polyprotein | Botrylloides leachii nidovirus |
| nsp10 | pp1a | Bovine nidovirus TCH5 |
| nsp8 | replicase polyprotein 1ab | Chinook salmon bafinivirus |
| nucleocapsid | capsid protein | California sea lion astrovirus 2 |

**S5 Table- Delta-BLAST results for negative sense ssRNA viruses**

| SARS-CoV-2 | Paramyxoviridae | Virus name |
| --- | --- | --- |
| Spike | Fusion protein | Avian metaavulavirus 20 |
| helicase | Large protein | Simian orthorubulavirus |
| ORF7a | Large polymerase protein | Avian paramyxovirus 14 |
| nsp11 | Hemagglutinin-neuraminidase | Human parainfluenza virus 4a |

| SARS-CoV-2 | Rhabdoviridae | Virus name |
| --- | --- | --- |
| 2’-O-ribose methyltransfrase | L polymerase protein | Vesicular stomatitis Indiana virus |
| ORF7a protein | RdRp | Wenling crustacean virus 11 |

| SARS-CoV-2 | Filoviridae | Virus name |
| --- | --- | --- |
| 2’-O-ribose methyltransfrase | RdRp | Bombali ebolavirus |

**S6 Table- B cell epitopes associated with neutralizing antibodies**

| IEDB Epitope ID | Description | Starting Position | Ending Position | Antigen Name |
| --- | --- | --- | --- | --- |
| 1312699 | IGVTQNVLYENQKLI | 909 | 923 | surface glycoprotein [SARS-CoV2] |
| 1313930 | VTYVPAQEKNFTTAP | 1065 | 1079 | surface glycoprotein [SARS-CoV2] |
| 1334487 | VTYVPAQEKNFTTAP + GLUC(N10) | 1065 | 1079 | surface glycoprotein [SARS-CoV2] |

**S7 Table- T cell epitopes associated with cytokine production**

| IEDB Epitope ID | Description | Starting Position | Ending Position | Antigen Name |
| --- | --- | --- | --- | --- |
| 2801 | ALNTLVKQL | 958 | 966 | surface glycoprotein [SARS-CoV2] |
| 23293 | GWTFGAGAALQIPFA | 885 | 899 | surface glycoprotein [SARS-CoV2] |
| 54507 | RLDKVEAEV | 983 | 991 | surface glycoprotein [SARS-CoV2] |
| 63951 | TGRLQSLQTYVTQQL | 998 | 1012 | surface glycoprotein [SARS-CoV2] |
| 69657 | VLNDILSRL | 976 | 984 | surface glycoprotein [SARS-CoV2] |
| 1069576 | FTTAPAICHDGKAHF | 1075 | 1089 | surface glycoprotein [SARS-CoV2] |
| 1070803 | KPSKRSFIEDLLFNK | 811 | 825 | surface glycoprotein [SARS-CoV2] |
| 1071768 | PHGVVFLHVTYVPAQ | 1057 | 1071 | surface glycoprotein [SARS-CoV2] |
| 1071978 | QMAYRFNGIGVTQNV | 901 | 915 | surface glycoprotein [SARS-CoV2] |
| 1072604 | SFPQSAPHGVVFLHV | 1051 | 1065 | surface glycoprotein [SARS-CoV2] |
| 1073938 | VQIDRLITGRLQSLQ | 991 | 1005 | surface glycoprotein [SARS-CoV2] |
| 1309913 | PFAMQMAYRFNGIGV | 897 | 911 | surface glycoprotein [SARS-CoV2] |
| 1310281 | APHGVVFLHVTYVPA | 1056 | 1070 | surface glycoprotein [SARS-CoV2] |
| 1310282 | AQALNTLVKQLSSNF | 956 | 970 | surface glycoprotein [SARS-CoV2] |
| 1310303 | CAQKFNGLTVLPPLL | 851 | 865 | surface glycoprotein [SARS-CoV2] |
| 1310415 | FNGLTVLPPLLTDEM | 855 | 869 | surface glycoprotein [SARS-CoV2] |
| 1310434 | GAISSVLNDILSRLD | 971 | 985 | surface glycoprotein [SARS-CoV2] |
| 1310503 | IPFAMQMAYRFNGIG | 896 | 910 | surface glycoprotein [SARS-CoV2] |
| 1310542 | KLIANQFNSAIGKIQ | 921 | 935 | surface glycoprotein [SARS-CoV2] |
| 1310586 | LITGRLQSLQTYVTQ | 996 | 1010 | surface glycoprotein [SARS-CoV2] |
| 1310593 | LLFNKVTLADAGFIK | 821 | 835 | surface glycoprotein [SARS-CoV2] |
| 1310623 | LTDEMIAQY | 865 | 873 | surface glycoprotein [SARS-CoV2] |
| 1310796 | SFIEDLLFNKVTLAD | 816 | 830 | surface glycoprotein [SARS-CoV2] |
| 1310852 | TLVKQLSSNFGAISS | 961 | 975 | surface glycoprotein [SARS-CoV2] |
| 1310947 | WTFGAGAALQIPFAM | 886 | 900 | surface glycoprotein [SARS-CoV2] |
| 1313359 | QLSSNFGAISSVLND | 965 | 979 | surface glycoprotein [SARS-CoV2] |
| 1313363 | QNVLYENQKLIANQF | 913 | 927 | surface glycoprotein [SARS-CoV2] |
| 1313987 | YENQKLIANQFNSAI | 917 | 931 | surface glycoprotein [SARS-CoV2] |
| 1316310 | FAMQMAYRF | 898 | 906 | surface glycoprotein [SARS-CoV2] |
| 1316853 | FPQSAPHGV | 1052 | 1060 | surface glycoprotein [SARS-CoV2] |
| 1329348 | ENQKLIANQFNSAIGKI | 918 | 934 | surface glycoprotein [SARS-CoV2] |
| 1329820 | NQNAQALNTLVKQLSSNFG | 953 | 971 | surface glycoprotein [SARS-CoV2] |
| 1330164 | TDEMIAQYTSALLA | 866 | 879 | surface glycoprotein [SARS-CoV2] |
| 1330361 | YENQKLIANQFNSAIGKIQ | 917 | 935 | surface glycoprotein [SARS-CoV2] |
| 1370489 | SKRSFIEDLLFNKVTLA | 813 | 829 | surface glycoprotein [SARS-CoV2] |
| 1392110 | DLLFNKVTL | 820 | 828 | surface glycoprotein [SARS-CoV2] |
| 1392244 | KPSKRSFIEDLLFNKVTLAD | 811 | 830 | surface glycoprotein [SARS-CoV2] |
| 1392420 | SFPQSAPHGVVFLHVTYVPA | 1051 | 1070 | surface glycoprotein [SARS-CoV2] |
| 1393965 | NTLVKQLSSNFGAISSV | 960 | 976 | surface glycoprotein [SARS-CoV2] |
| 1397094 | ANQFNSAIGKIQDSL | 924 | 938 | surface glycoprotein [SARS-CoV2] |
| 1397135 | GKGYHLMSFPQSAPH | 1044 | 1058 | surface glycoprotein [SARS-CoV2] |
| 1397188 | QIPFAMQMAYRFNGIGV | 895 | 911 | surface glycoprotein [SARS-CoV2] |
| 1397189 | QKLIANQFNSAIGKI | 920 | 934 | surface glycoprotein [SARS-CoV2] |
| 1410559 | ALQIPFAMQMAYRFNGIGV | 893 | 911 | surface glycoprotein [SARS-CoV2] |
| 1505853 | SKRSFIEDLLFNKVTLADA | 813 | 831 | surface glycoprotein [SARS-CoV2] |

**S7 table - Continued**

**S8 Table- Representative ssRNA viruses used for phylogenetic analyses – Continued on the next page**

| **ssRNA sense** | **Family** | **Virus** |
| --- | --- | --- |
| Negative | Orthomyxoviridae | Influenza virus A |
| Negative | Orthomyxoviridae | Influenza virus B |
| Negative | Orthomyxoviridae | Influenza virus C |
| Negative | Orthomyxoviridae | Influenza virus D |
| Negative | Orthomyxoviridae | Isavirus |
| Negative | Paramyxoviridae | Measles morbillivirus |
| Negative | Paramyxoviridae | Rinderpest morbillivirus |
| Negative | Paramyxoviridae | Mumps rubulavirus |
| Negative | Paramyxoviridae | Parainfluenza virus 1 |
| Negative | Paramyxoviridae | Parainfluenza virus 2 |
| Negative | Paramyxoviridae | Parainfluenza virus 3 |
| Negative | Paramyxoviridae | Parainfluenza virus 5 |
| Negative | Filoviridae | Ebola virus zaire |
| Negative | Filoviridae | Marburg virus |
| Negative | Filoviridae | Cuevavirus |
| Negative | Filoviridae | Dianlovirus |
| Negative | Rhabdoviridae | Rabies virus |
| Negative | Rhabdoviridae | Vesicular stomatitis virus |
| Negative | Rhabdoviridae | European bat lyssa virus 1 |
| Negative | Rhabdoviridae | Bas congo virus |
| Negative | Rhabdoviridae | Piscine novirhabdovirus |

| **Sense** | **Family**  **S8 table - Continued** | **Virus** |
| --- | --- | --- |
| Positive | Coronaviridae | SARS-COV2 |
| Positive | Coronaviridae | SARS |
| Positive | Coronaviridae | Bat-MERS like coronavirus |
| Positive | Coronaviridae | Human OC43 |
| Positive | Coronaviridae | Human HKU1 |
| Positive | Coronaviridae | PEDV |
| Positive | Coronaviridae | Murine hepatitis virus |
| Positive | Picornaviridae | Enterovirus, EV-71 |
| Positive | Picornaviridae | Enterovirus, Coxackie virus |
| Positive | Picornaviridae | Enterovirus, poliovirus |
| Positive | Picornaviridae | Enterovirus, Rhinovirus A |
| Positive | Picornaviridae | Enterovirus B |
| Positive | Picornaviridae | Hepatovirus A, hep A virus |
| Positive | Picornaviridae | Aphthovirus |
| Positive | Flaviviridae | Hepatitis C virus |
| Positive | Flaviviridae | Zika virus |
| Positive | Flaviviridae | Dengue virus |
| Positive | Flaviviridae | West Nile virus |
| Positive | Flaviviridae | Bovine viral diarrhea virus 1 |
| Positive | Toga | Semliki Forest virus |
| Positive | Toga | Sindbis virus |
| Positive | Toga | Eastern equine encephalitis virus |
| Positive | Toga | Western equine encephalitis virus |
| Positive | Toga | Rubella virus |
| Positive | Calici | Norovirus |
| Positive | Calici | Feline calicivirus |
| Positive | Calici | Sapovirus |
| Positive | Calici | Primate norovirus |
| Positive | Calici | Canine vesivirus |
| Positive | Calici | Norwalk like virus |

**S8 table - Continued**
